# Short term body mass manipulation in powerlifting: a narrative review and best practice recommendations

**DOI:** 10.1080/15502783.2025.2591783

**Published:** 2025-11-30

**Authors:** Alexander Renner, Eric R. Helms, Kedric Kwan, Robert Csapo

**Affiliations:** a Centre for Sports Science University Sports, University of Vienna, Vienna, Austria; b Sport Performance Research Institute New Zealand (SPRINZ), Auckland University of Technology, Auckland, New Zealand; c Department of Exercise Science and Health Promotion, Muscle Physiology Laboratory, Florida Atlantic University, Boca Raton, FL, United States

**Keywords:** Short term body mass manipulation, making weight, weight cutting, rapid weight loss, powerlifting

## Abstract

Short Term Body Mass Manipulation (SBM) is frequently used in powerlifting by athletes to qualify for lower weight classes and improve relative competitiveness. The three primary physiological pathways that SBM leverages are gastrointestinal content reduction, glycogen storage and body water manipulation, in addition to post-weigh-in refueling. Despite its high prevalence among athletes competing in the International Powerlifting Federation (IPF), the scientific literature on SBM remains limited, and sport-specific guidelines are currently lacking. This narrative review summarizes the current evidence on SBM in powerlifting, with a focus on physiological mechanisms, practical implementation, and associated risks. The specific demands of IPF competition, consisting of maximal strength performance after only a two-hour window between weigh-in and competition, necessitate uniquely tailored SBM strategies. SBM should not be regarded as a standard preparation method. Instead, it should be seen as a targeted intervention to be applied with caution and strategic intent. The decision to implement SBM must be based on individual assessment, physiological plausibility, and a well-considered cost–benefit rationale. Ensuring effective rehydration and refueling between weigh-in and competition is critical to support both safety and performance. This review provides sport specific, evidence-based recommendations to assist practitioners in applying SBM responsibly within the context of powerlifting.

## 1. Introduction

Powerlifting is a weight-class sport that requires athletes to weigh in within their class limits. This means being above the upper boundary of the next lower class and at or below the upper limit of their chosen class. Since many athletes exceed these limits during training or the off-season, the majority of powerlifters use strategies in the days before competition to acutely manipulate body mass and qualify [1].

Commonly referred to as weight cutting, rapid weight loss, or making weight, this practice is herein described using the term Short Term Body Mass Manipulation (SBM). This terminology is favored for several reasons. First, commonly used expressions often lack precision, as they do not clearly distinguish between acute and chronic body mass changes. Second, most colloquial terms focus exclusively on reduction, whereas short-term manipulation also encompasses the post-weigh-in refueling process, which typically results in a body mass increase between the weigh-in and competition. In principle, SBM can also involve deliberately increasing body mass to qualify for a higher weight class, although this is very uncommon, as gaining weight largely occurs over longer time frames. Third, the term body mass is scientifically more accurate than body weight, since mass, measured in kilograms, is the parameter relevant for competition, whereas weight is a force measured in newtons.

Various SBM practices are associated with risks to both performance and health [2–6]. Moreover, the need for sport-specific recommendations is repeatedly emphasized in the scientific literature. This is due to the unique weigh-in procedures and metabolic demands across different sports [2,7,8].

In the International Powerlifting Federation (IPF), the weigh-in period begins two hours before the start of competition [9]. This is significantly shorter than in many other sports, where weigh-ins may occur up to 24 hours in advance [4,8]. The sport’s emphasis on maximal strength in the squat, bench press, and deadlift further underscores the limitations of generalized SBM strategies, where the focus might be on preserving endurance performance [2,8,10]. The combination of the close weigh-in to competition and the focus on maximal strength means that generalized SBM recommendations often fail to meet the specific needs of powerlifting athletes.

A further complication is that powerlifters often rely on informal sources of information, including coaches, peers and online material [1]. This may, in part, be attributed to the current absence of evidence-based recommendations on SBM specifically developed for powerlifting.

This review aims to fill this research gap of evidence-based recommendations for SBM in powerlifting by synthesizing the current literature, examining weight class selection, the prevalence and practices of SBM in the sport, and the physiological mechanisms and practical methods involved. It further addresses strategies for refueling between weigh-in and competition, considerations unique to female athletes, and the associated risks, limitations, and ethical concerns, culminating in sport specific, evidence-based guidance for the safe and strategic implementation of SBM in competitive powerlifting.

## 2. Weight class selection and long-term preparation

Powerlifting uses fixed weight classes. Therefore, the optimal class for an individual athlete may not be obvious. As an example, an 88 kg athlete might compete lighter relative to the rest of the 93 kg class, gradually gain weight to fit that class, or cut to 83 kg via sustained caloric deficit and possibly SBM. The best approach depends on factors like personal preference, current and projected body composition relative to habitual body composition, competitive context, long-term development, individual physiological factors related to metabolism and body composition, and health.

As a first step in determining the most appropriate weight class, a comprehensive evaluation of the athlete’s current body composition and health status should be conducted. This includes assessing body fat percentage, lean body mass, and overall anthropometric profile [11]. In addition, general health markers such as blood pressure, lipid profile, and insulin sensitivity should be considered, particularly if significant body mass changes are being contemplated as these body mass changes could contribute to an improvement or worsening of the athletes health status [12–17].

It is essential to estimate what the athlete’s body composition would be in either a higher or lower weight class. Would an increase in body mass primarily result in additional muscle, or would it predominantly be fat gain? Conversely, would a reduction in body weight preserve lean mass, or would it lead to undesirable muscle loss? Importantly, individuals with lower baseline adiposity tend to lose a greater proportion of fat-free mass relative to total body mass during weight loss [18]. A key concern in such cases is whether the athlete would need to reach an unreasonably low level of body fat to make weight, which may compromise both performance and physiological function. The dual intervention point model of body fat regulation proposes that there are theoretical lower limits of adiposity, below which homeostatic adaptations occur, including reductions in total daily energy expenditure and increases in hunger, which make sustaining such levels difficult [19]. If prolonged, these adaptations may mimic low energy availability and lead to symptoms associated with Relative Energy Deficiency in Sport (RED-S) [20]. Regular body composition assessments support weight class selection by tracking the athlete’s rate of lean mass gain and estimating how much body fat can be lost before approaching unsustainable levels. Ultimately, what constitutes a reasonable and sustainable degree of leanness varies between individuals and is shaped by two overlapping factors: the size of the energy deficit and the individual’s baseline body fat relative to their physiologically maintainable range [18,19]. These considerations are critical not only for optimizing performance but also for safeguarding long-term health [21].

Equally important is the athlete’s personal goal. Does the athlete express a desire to gain, maintain, or lose weight? Their motivation and psychological readiness for a bodyweight change must be factored into the decision-making process. Additionally, the competitive context must be analyzed. At which bodyweight does the athlete have the best chance of achieving competitive success, such as qualifying totals, podium placements, or records?

From a long-term athletic development perspective, it should also be considered that skeletal muscle mass is a primary determinant of strength performance in powerlifting [22–24]. Furthermore, the process of gaining skeletal muscle mass and strength occurs at an increasingly slower rate as athletes develop more skeletal muscle mass and strength over time, following roughly a linear-log relationship [25–27]. Finally, an energy surplus, while not required to build skeletal muscle, can increase rates of muscle gain [28] and large enough energy deficits can slow these rates [29]. Therefore, competing at a lower bodyweight may limit the athlete’s capacity for hypertrophy and, by extension, their long-term performance ceiling. In contrast, a higher weight class may offer more favorable conditions for muscle growth, provided it aligns with health and competitive goals.

In summary, selecting the most appropriate weight class in powerlifting requires an individualized, multifactorial approach. Athletes and coaches must balance health status, body composition, performance potential, and personal preferences, while also considering both short-term competitiveness and long-term athletic development. Strategic use of SBM can support weight class transitions. However, the foundation should always be well managed long term caloric intake. Ultimately, the chosen weight class should enable the athlete to train, recover, and perform at their highest sustainable level.

## 3. Prevalence and practices of SBM in powerlifting

Despite the scarcity of sport-specific literature, a small body of survey-based research provides insight into the prevalence, magnitude, and methods of SBM in powerlifting.

Across three studies involving lifters competing within the IPF, the reported prevalence of SBM was approximately 87.4%. Among these athletes, the average acute body mass reduction prior to competition was 3.2%, with individual values ranging widely. The most common strategy was manipulation of body water balance [1,3,5].

Female athletes reported slightly higher maximal historical body mass losses than their male counterparts [1,3]. Notably, in a sample of lifters at the 2018 IPF Classic World Championship, 90% of medal winners reported engaging in SBM, compared to 70% of non-medalists. Medalists also reported larger average reductions (4.1% vs. 2.7%) and higher maximal historical losses [5].

A study on youth lifters aged 14–18 reported that 68% engaged in dehydration-based strategies. However, no data were provided on the extent of body mass loss. General knowledge regarding safe and effective SBM methods was low [30].

Across studies, negative physical and psychological experiences were commonly reported, including fatigue, discomfort, mood disturbances, anxiety, and isolation [3,5]. Athletes primarily relied on informal sources of guidance, such as coaches, peers and online material [1].

In summary, SBM appears to be a highly prevalent practice among competitive powerlifters competing within the IPF. Most athletes lose ~3% of their body mass, typically through body water balance manipulation, though substantial interindividual variability exists.

## 4. Mechanisms and methods

Multiple studies identify three primary physiological pathways for SBM: gastrointestinal content reduction, glycogen storage manipulation, and body water balance manipulation [2,7,8]. The following sections review the physiology and current research on each pathway in the context of SBM in powerlifting.

### 4.1. Gastrointestinal content reduction

Nolan et al. (2022) reported that 56.5% of powerlifters have engaged in gastrointestinal content reduction at some point in their career. This strategy is theorized to acutely lower body mass by reducing the volume and mass of digestive matter within the gastrointestinal tract, without affecting hydration or energy availability [2,7,8,31]. The key nutritional target is dietary fiber, alongside reduced food volume. Insoluble fiber increases bulk and transit speed, while soluble fiber enhances viscosity and water retention in the gut [32,33]. Given that whole-gut transit times generally range from 10 to 73 hours [34],it is hypothesized that fiber restriction can lead to measurable weight loss within a few days. Notably,however,foods high in soluble fiber specifically draw water into the gut and increase stool hydration [35], meaning that soluble fiber reduction does reduce body water, but from a specific compartment (the gastrointestinal tract) that is unlikely to impact performance.

Reducing gastrointestinal content is often recommended as a first-line SBM strategy [7]. Foo et al. (2022) conducted a controlled trial with 19 healthy males who switched from a high-fiber diet of about 30 grams per day to a low-fiber diet with less than 10 grams. Energy and macronutrient intake remained constant. After four days, body mass dropped slightly but significantly (−0.40%), with a further reduction by day five (−0.74%). Participants reported more hunger and changes in bowel habits, but the intervention was generally well tolerated. Variability in body mass change may relate to individual differences in gut motility, which tends to be slower in females and older adults [34,36]. As the study included only younger males, it’s unclear if the findings apply to females and older adults. More research is needed to assess sex- and age-related differences.

Although clinical bowel preparations like laxatives can reduce intestinal content, they may impair performance [37] and raise anti-doping concerns, as some are banned in competition. Thus, pharmacological bowel clearance is not recommended. Dietary strategies such as low-fiber diets are safer, more practical, and may preserve performance. Nonetheless, laxatives remain in use among powerlifters [1,3,5].

Within powerlifting, gastrointestinal content reduction is commonly referred to as a “gut cut”. A commonly promoted version of this approach involves a diet centered around nuts, protein shakes, and high-sugar foods such as candy or ice cream. The underlying idea is to achieve high caloric density while reducing the overall mass and volume of gastrointestinal contents. However, this approach overlooks several key physiological considerations.

First, as previously discussed, dietary fiber is the key nutrient for SBM using gastrointestinal content reduction, and nuts are typically high in fiber [38,39]. Therefore, consuming large quantities of nuts is counterproductive to the goal of reducing gastrointestinal content. Second, while high sugar foods such as candy and ice cream are more energy-dense than low fiber starchy foods, this is largely due to the latter's higher water content [40–45]. Since water consumed through solid foods contributes to total body water balance [46],there is no physiological advantage to replacing low fiber staples like white rice or bread with high sugar foods as any reduction in water intake from food by choosing high-sugar items over low fiber starchy foods must be compensated by drinking more fluids to maintain water balance. Finally,this practice often leads to a disproportionate increase in dietary fat intake while simultaneously reducing carbohydrate consumption [47]. Consequently, any observed decrease in body mass is likely attributable, at least in part, to glycogen depletion due to lower carbohydrate availability, or to dehydration. The latter may result from the fact that high sugar foods and nuts generally contain less water than low fiber starchy foods. If fluid intake remains constant, this dietary substitution may reduce overall hydration status. Thus, the weight loss achieved is likely in part the result of glycogen depletion or dehydration, rather than a true reduction in gastrointestinal content, thereby undermining the intended purpose.

Given current evidence, dietary reduction of gastrointestinal content should be a first-line SBM method. Athletes consuming more than 30 g of fiber per day, as recommended by sports nutrition guidelines [48],may lose approximately 0.5% to 2% of body mass within 48 to 96 hours on a diet containing less than 10 g of fiber per day [31]. The magnitude and timing of weight loss may depend on baseline fiber intake, dietary fiber composition and individual transit times. To minimize performance impact, low-fiber diets should include starchy foods like white rice and bread, as well as low-fiber protein and fat sources such as eggs, dairy, meat, and oils, rather than high-fat or high-sugar options. If used as the sole SBM method, macronutrient intake should match the habitual diet. If combined with glycogen depletion, carbohydrate intake will naturally decrease.

### 4.2. Glycogen storage manipulation

Glycogen and its associated water can contribute substantially to total body mass [7,49,50]. Therefore, depleting the body's glycogen stores may theoretically result in a notable short-term reduction in body mass.

Two primary methods can deplete glycogen, either used separately or in combination. These are reduced or eliminated carbohydrate intake and physical activity. Carbohydrate restriction, either by avoiding carbohydrate-rich foods or through fasting, leads to gradual glycogen depletion over several days. When choosing a low carbohydrate diet, one must be aware of potential negative symptoms and the underlying physiology, as part of the body mass loss is likely due to adjusted body water levels. The decrease in insulin caused by carbohydrate restriction increases renal sodium and potassium excretion, which in turn promotes diuresis and lowers body water levels [51]. Additionally, the water associated with glycogen should not be regarded as a meaningful buffer against dehydration during glycogen depletion [52]. Exercise accelerates glycogen depletion by using glycogen as fuel [2,7]. Resistance training causes region- and fiber type-specific depletion, particularly in type 2 fibers. This can potentially lead to fatigue and reduced power output [53].

Choosing a method depends on sport-specific and individual tapering practices. For athletes unaccustomed to high metabolic demand, exercise-based depletion is inadvisable during competition week, when recovery is the focus; hence, gradual dietary restriction is preferred. Well-adapted athletes may use glycogen depleting exercise if it aligns with their tapering practices [7,50,54].

Another relevant consideration is the metabolic demand of the sport. While many sports are limited by available glycogen, powerlifting is characterized by maximal strength efforts that typically last less than 10 seconds. These efforts are primarily anaerobic and alactic, relying on the breakdown of stored phosphagens such as ATP and phosphocreatine, rather than glycogen [55]. Multiple studies indicate both short-term and longer-term reductions in carbohydrate intake (up to three months) do not significantly impair maximal strength [56–58]. Overall, it appears unlikely that carbohydrate availability or saturated glycogen stores play a major role in strength performance [59]. Consequently, short-term reductions in carbohydrate intake do not seem to negatively affect strength levels. However, it is important to note that carbohydrates and glycogen availability may play a more pronounced role in high-volume or high-repetition strength training sessions, where the reliance on glycolytic energy pathways is greater [60]. That said, carbohydrate supplementation does not appear to enhance performance in typical low-volume strength training contexts [61]. Lastly, the timeframe available for glycogen storage replenishing following weigh-in should be considered when designing a glycogen depletion strategy. In sports with a 24-hour weigh-in period, athletes have ample time to replenish glycogen stores prior to competition. In contrast, powerlifting typically features a 2-hour weigh-in, which offers a much narrower window for effective glycogen restoration.

Glycogen storage and its depletion should be viewed as a continuum. Glycogen stores are rarely either fully saturated or completely depleted [54]. Although glycogen depletion does not appear to significantly affect maximal strength, athlete wellbeing should remain a central consideration. If glycogen storage depletion is chosen for SBM, a reduction in habitual carbohydrate intake for 3 to 7 days is recommended with maintained protein and fat intake. This reduction in carbohydrate and overall energy intake, while resulting in short term body mass reductions, may also increase hunger, fatigue, and compromise athlete wellbeing. Glycogen depletion workouts are generally not recommended in powerlifting, though their appropriateness may vary depending on the individual athlete. The amount of body mass that can be lost through glycogen depletion appears to be highly individual and dependent on several factors, including habitual carbohydrate intake, initial glycogen stores, the extent of dietary carbohydrate reduction, and the level of metabolic demand during the depletion period. Current evidence suggests that, depending on these factors, glycogen depletion and the associated loss of bound water can lead to a reduction in body mass of around 2% [50,57].

### 4.3. Body water balance manipulation

Among powerlifting athletes, manipulation of body water balance represents both the most prevalent and most effective SBM strategy [1,3,5,30]. Owing to the high proportion of water in the human body, dehydration can induce rapid and substantial reductions in body mass. While effective, this method risks adverse health outcomes and performance impairments [2,4].

Body mass loss through body water manipulation is typically achieved by reducing or ceasing fluid intake prior to weigh-in. In many cases, this phase of fluid restriction is preceded by a period of increased fluid intake, a strategy known as water loading. The purpose of water loading is to temporarily increase urine production. When fluid intake abruptly decreases following loading, fluid excretion stays high for a short period, resulting in a transient negative fluid balance and greater loss of body mass (~0.8%) than fluid restriction alone [62]. Further body mass reduction can be achieved through passive or active dehydration methods. Passive methods typically involve heat exposure to induce sweating, such as sauna use, hot baths, sweating suits, or excessive clothing. Active methods primarily rely on exercise induced sweating. Finally, some athletes induce salivary fluid loss via spitting into a cup, usually facilitated by stimulating saliva production through chewing gum or spice.

Reducing sodium intake may further enhance water loss. In hypertensive individuals, a five-day low-sodium diet results in greater reductions in body mass compared to normal or high-sodium diets [63]. However, the specific contribution of sodium restriction to SBM has not been directly investigated.

Body water manipulation may reportedly reduce body mass by as much as 10% [64]. However, when substantial body mass reductions are achieved through dehydration, adverse health outcomes, performance impairments, and the effectiveness of rehydration after weigh-in become major concerns.

Dehydration can adversely impact cardiovascular, neurological, renal, gastrointestinal, and musculoskeletal health [65]. Extreme dehydration reduces plasma and stroke volume, elevate heart rate, and impair arteriovenous oxygen difference during submaximal exercise. These changes may reduce renal perfusion, cause electrolyte disturbances, increase susceptibility to heat-related illness, induce muscle cramping, and increase exercise-induced nausea [16,66]. Even after body mass is restored post-weigh-in, urine osmolality and specific gravity often indicate persistent dehydration [10]. There is growing concern that dehydration-induced SBM via dehydration can cause acute kidney injury, as elevated levels of creatinine, blood urea nitrogen, and urine specific gravity can persist during or following periods of rapid body mass reduction [67]. Importantly, acute kidney injury and chronic kidney disease are closely linked and may influence each other [68]. A major concern with water loading is hyponatremia. Common water loading protocols couple an initial increase in sodium with minimal sodium consumption during the end of water loading and restriction. While moderate protocols, as used by Reale et al. (2018), did not induce hyponatremia, extreme water-loading protocols combining well over 100 mL/kg body mass per day with sodium restriction may increase risk [62,69–71].

Dehydration negatively impacts maximal strength. However, if followed by rehydration, these negative effects may be mitigated. Schoffstall et al. (2001) reported a passive sweating-induced dehydration of 1.5% body mass decreased maximal bench press strength by 5.6% in strength-trained males, but a two-hour rest period with water consumption reversed these effects [72]. Gann et al. (2001) observed dehydration-induced 3% body mass reductions in strength-trained females significantly reduced maximal bench press strength and perceived recovery. However, maximal leg press strength was not significantly affected, nor the number of bench and leg press sets to failure at 75% 1-RM, vertical jump, or ratings of perceived exertion. Notably, the control group also lost body mass through heat exposure but was provided adequate rehydration time [73]. Judelson et al. (2007) suggest dehydration-induced body mass losses of 3–4% can reduce strength by ~2%, although only 15 of 70 reviewed findings were statistically significant. Importantly, no studies included powerlifting-specific exercises, limiting the direct applicability of these findings [74]. Similarly, Savoie et al. (2015) reported significant reductions in muscle strength of ~5.5% following dehydration, noting no significant differences between upper-body and lower-body strength losses [75]. Furthermore, despite a three-hour recovery period, Barley et al. (2018) observed impaired muscular strength-endurance and increased perceived fatigue following a dehydration-induced body mass reduction of 3.2%, without changes in central or peripheral physiological markers. Therefore, altered fatigue perception may primarily underly dehydration-induced performance decrements [76].

Based on the current evidence dehydration should be limited to what can be fully replenished between weigh-in and competition, making rehydration a central concern. In the case of experienced athletes, where physiological, psychological, and performance data are available, more aggressive manipulation may be considered, but generally not in excess of 3% body mass, which is proposed as an upper limit following a two hour weigh-in [7].

### 4.4. Combination of multiple mechanisms

The only original study to date investigating combined SBM strategies in a powerlifting context is by Matras et al. (2025). Competitive male powerlifters were assigned to either a structured SBM protocol or a control group. The protocol included an energy deficit of approximately 10%, a low-fiber ketogenic diet, aggressive fluid and sodium restriction, and concluded with a rehydration strategy. Over 14 days, both groups underwent two simulated competitions. The intervention group reduced body mass by an average of 4.81%, with individual reductions from 0.58% to 8.10%, while strength remained stable. Relative strength improved, and dehydration was effectively managed through rehydration. The high individual variability highlights the unpredictable nature of SBM without individualized reference values, emphasizing the need for prior experience and tailored protocols. This investigation showed that body mass reductions around 5% can be feasible without impairing strength, although real-world application remains context and individual specific due to the controlled setting [77].

Reale et al. (2018) also investigated the effect of water loading on SBM in combat sport athletes alongside additional SBM methods. Before water loading, the experimental and control groups restricted energy and dietary fiber (to 10–13 g/day), which reduced body mass 1–2% prior to fluid restriction. Across five days, 3.2% and 2.4% mean body mass reductions occurred in the water loading and control groups, respectively. No significant reductions nor differences in physical performance within or between groups were observed [62].

Fasting, the cessation of food and fluid intake typically starting the day of or evening before weigh-in, influences gastrointestinal contents, glycogen, and hydration [2]. Body mass lost during this period is difficult to predict, depending on temperature, humidity, initial body mass, body temperature, and metabolism. Often unaddressed in SBM research, fasting is not recommended as a primary strategy but can be used in athletes slightly above target weight following the primary SBM strategy.

A modified version, known as competition day scale fasting, involves the athlete stepping onto the scale while holding any food or drink they intend to consume afterward. If the total mass remains within the weight class limit, consumption is permitted. This method offers a pragmatic and effective strategy for managing weigh-in day nutrition.

## 5. Refueling after weigh-in

Following a successful weigh-in, refueling should commence immediately, especially after substantial body mass loss. The urgency and complexity of refueling increases proportionally with the magnitude of body mass reduction, especially when dehydration is the primary SBM strategy. In such instances, rapid and efficient rehydration is required, but is constrained by gastrointestinal tolerance and fluid absorption capacity, typically ranging from 1 to 1.5 liters per hour [70].

To restore fluid balance, athletes should consume ~125% to 150% of the body mass lost through dehydration [70,78–80]. Given the upper limit of fluid absorption and the standard two-hour post-weigh-in period, dehydration-induced losses should generally not exceed ~2 kg to facilitate timely rehydration.

Rehydration beverages should contain ∼1700 mg of sodium, ∼800 mg of potassium, ∼2300 mg of chloride and ∼13 g of carbohydrates per liter. This composition resembles medical-grade oral rehydration solutions designed to maximize fluid absorption and retention. However, if the athlete prefers consuming carbohydrates in liquid rather than solid form, the carbohydrate content may be increased, making the beverage resemble more a sports drink [81]. Consuming 750–1000 mL of either solution immediately post weigh-in can initiate rehydration effectively. To mitigate gastrointestinal discomfort risk, solid food intake should be postponed ~ 30 minutes following initial fluid ingestion [82].

If tolerated, a small, digestible meal with ~50–100 g carbohydrates can follow after 30 minutes with continued fluid intake at the 1–1.5 L/hour absorption rate. Total carbohydrate intake from liquid and solid sources post-weigh-in should consider comfort and preference. Up to ~60 g of glucose can be absorbed per hour; combining glucose with fructose allows absorption of up to ~90 g/hour [81].

Small amounts of protein and fat may be included based on individual preference to support satiety and wellbeing. During extended competition (i.e. during bench press and deadlifts), further intake of fluids and carbohydrates should be based on subjective comfort, allowing athletes to eat and drink as tolerated.

## 6. Considerations for the female athlete

While the underlying physiological principles are the same for male and female athletes, certain considerations may be more pertinent to female athletes.

Relative Energy Deficiency in Sport (RED-S), formerly termed the Female Athlete Triad, can affect males, but remains most prevalent among young females. It describes a syndrome of impaired physiological function, including but not limited to disruptions in metabolic rate, menstrual function, bone health, immune function, protein synthesis, and cardiovascular health, caused by insufficient energy availability [83]. In healthy athletes, energy intake adequately supports training and physiological function. However, chronically low energy availability due to dieting, disordered eating, or increased energy expenditure can cause multisystemic adaptations that prioritize survival over performance [84]. Accordingly, athletes should aim to compete in a weight class that permits adequate fueling to reduce risk of RED-S. It is not advisable to pursue a weight class that necessitates decreasing body fat below ~12% in female athletes [85]. However, adverse outcomes associated with RED-S can occur in female athletes exposed to chronic low energy availability even when body fat percentage is 12% to 22% [86].

The menstrual cycle can also influence hydration and carbohydrate metabolism. Although estrogen and progesterone fluctuate throughout the menstrual cycle, current evidence does not support a consistent effect of these hormonal changes on fluid retention, sweat rate, dehydration risk, or post-exercise rehydration [87]. However, most research on dehydration has been conducted in males. Further studies to understand the interaction between female sex hormone variation and hydration status are needed [88]. Core body temperature increases by 0.3 °C to 0.7 °C during the progesterone-dominant luteal phase, reflecting enhanced heat conservation, and is lower during the estrogen-dominant follicular phase, when heat dissipation increases [89]. These hormonal shifts however do not consistently alter thermoregulatory responses during exercise [87]. Nonetheless caution is warranted, and core temperature should be monitored during active or passive sweating protocols [86]. Despite the lack of experimental evidence, many women report perceived changes in fluid retention. These perceptions often involve bloating, most commonly around menstruation onset. This suggests a disconnect between subjective experiences and measurable physiological outcomes [87]. Increased carbohydrate intake during the luteal phase has been recommended due to reduced gluconeogenesis during exercise caused by hormonal fluctuations [90]. However, this is unlikely to significantly impact SBM strategies in the context of powerlifting.

A further concern is the prevalence of body image related challenges associated with SBM, which may affect female athletes more frequently than males [91,92]. Pereira Vargas and Winter (2021) explored disordered eating experiences among female powerlifters, noting SBM was not solely used for competition but was closely connected to body image concerns. Lower weight classes were perceived as more consistent with socially desirable body ideals, illustrating a female-athlete paradox where athletes strive for performance while also conforming to societal beauty standards. The authors recommended that raising awareness and providing targeted education about disordered eating, SBM practices, nutrition and to improve body image (which may prevent disordered eating) is essential. Supporting this, Mancine et al. (2020) found a greater prevalence of disordered eating among female, but not mal, athletes participating in power sports, including powerlifting [91]. In contrast to acute SBM, recent evidence indicates a nine-week caloric deficit leading into competition may result in better weight satisfaction and well-being, without contributing to negative body image perceptions [93].

The primary consideration for female athletes engaging in SBM, as well as in long-term body mass reduction, is the prevention of RED-S. Although menstrual cycle fluctuations in body temperature and metabolism occur, current literature does not justify sex-specific SBM protocols. The decision to implement SBM should be based on performance, while openly acknowledging and addressing potential body image related challenges due to societal pressure to pursue or remain in lower weight classes.

## 7. Risks, limitations, and ethics

In addition to the previously discussed risks of SBM exceeding 3% body mass, additional impairments in high-intensity exercise performance and adverse effects on physical health can occur, including acute cardiovascular strain, hormonal imbalances, and suppressed immune function [10,94]. Many powerlifting athletes additionally report experiencing negative psychological effects when implementing SBM [3,5]. These psychological challenges should be carefully considered when determining whether SBM is appropriate.

Given these potential adverse effects, SBM warrants careful consideration guided by individualized assessment, supported by a clear cost-benefit rationale, and restricted to physiologically plausible magnitudes of body mass reduction. Whenever feasible, long-term body mass management should be prioritized instead. SBM is not appropriate for athletes under the age of 18 and individuals with medical contraindications such as a history of eating disorders, symptoms of RED-S, or kidney-related conditions.

Given the health and performance risks, the current prevalence of SBM among powerlifters appears disproportionately high. Rather than a routine preparatory strategy, SBM should be a specialized intervention reserved for experienced athletes with medical clearance, within 3% of their desired weight class, or, for highly experienced lifters, 5%, with realistic prospects of achieving meaningful competitive outcomes while being supported by experienced nutrition and health professionals. SBM should be avoided among novice athletes, regardless of age or performance level and SBM should be supervised by qualified professionals for safety. Powerlifting governing bodies should invest in continuous education for coaches and athletes to reduce SBM prevalence and promote safe, evidence-based practices.

## 8. Conclusion and recommendations

SBM is widely practiced in powerlifting. However, athletes’ understanding of its physiological basis, safety, and evidence-based application remains limited. Given the lack of scientific data, universal guidelines are premature. Due to its prevalence and limited athlete education, this review offers best practice recommendations tailored to powerlifting’s weigh-in procedures and performance demands. These recommendations may also apply to Olympic weightlifting, given similar competition formats and performance demands.

Athletes should first reduce body mass to within 3% of the target through a long-term caloric deficit. Acute strategies such as gastrointestinal content reduction, glycogen depletion, and dehydration can then be applied as needed. Combining methods distributes physiological stress and lowers the risk of performance decline. With adequate experience and individual data, reductions up to 5% may be feasible if no adverse effects occur.

Table 1 presents SBM protocols by target weight loss percentage. These reflect average outcomes, but individual variation is high. Athletes should develop personal profiles that track protocols used, body mass changes, performance, and wellbeing. This helps guide more precise and effective future strategies.

**Table 1.** SBM protocols depending on percentage of body mass reduction.

% Body mass	SBM methods	Concrete protocols
0%	• None	• Continue habitual diet and fluid intake
<0.5%	• Pre competition fasting, spitting if needed	• Cease food and fluid intake 1-6h pre weigh in
<1%	• Gastrointestinal content reduction • Pre competition fasting and spitting as needed	• If needed stimulate saliva flow via chewing gum or spice while spitting into a cup 30-60min pre weigh in or after first unsuccessful weigh in attempt • Low fiber (<10g/day) diet for 3−4 days pre competition (ensure habitual fiber intake > 30g/day) • Habitual carbohydrate intake • Habitual fluid intake • If needed completely cease food and fluid intake 1-6h pre weigh in • If needed stimulate saliva flow via chewing gum or spice while spitting into a cup 30-60min pre weigh in or after first unsuccessful weigh in attempt
<2%	• Gastrointestinal content reduction • Mild glycogen storage depletion • Pre competition fasting and spitting as needed	• Low fiber (<10g/day) diet for 3−4 days pre competition (ensure habitual fiber intake > 30g/day) • Low carbohydrate (<50g/day) diet for 2−3 days pre competition (ensure adequate habitual carbohydrate intake) • Habitual fluid intake • If needed completely cease food and fluid intake 1-6h pre weigh in • If needed stimulate saliva flow via chewing gum or spice while spitting into a cup 30-60min pre weigh in or after first unsuccessful weigh in attempt
<3%	• Gastrointestinal content reduction • Mild glycogen storage depletion • Mild body water balance manipulation • Pre competition fasting and spitting as needed	• Low fiber (<10g/day) diet for 3−4 days pre competition (ensure habitual fiber intake > 30g/day) • Low carbohydrate (<50g/day) diet for 2−3 days pre competition (ensure adequate habitual carbohydrate intake) • Habitual fluid intake, decrease fluid intake to 15−20 mL/kg body mass/day 10-24h pre weigh in, habitual sodium intake • If needed completely cease food and fluid intake 1-6h pre weigh in • If needed stimulate saliva flow via chewing gum or spice while spitting into a cup 30-60min pre weigh in or after first unsuccessful weigh in attempt
<4%	• Gastrointestinal content reduction • Severe glycogen storage depletion • Moderate body water balance manipulation • Pre competition fasting and spitting as needed	• Low fiber (<10g/day) diet for 3−4 days pre competition (ensure habitual fiber intake > 30g/day) • Low carbohydrate (<50g/day) diet for 4−7 days pre competition (ensure adequate habitual carbohydrate intake) • 60−90 mL/kg body mass/day 76-24h pre weigh in, 24 h pre weigh in 15 mL/kg body mass/day, habitual sodium intake • If needed completely cease food and fluid intake 1-6h pre weigh in • If needed stimulate saliva flow via chewing gum or spice while spitting into a cup 30-60min pre weigh in or after first unsuccessful weigh in attempt
<5%	• Gastrointestinal content reduction • Severe glycogen storage depletion • Severe body water balance manipulation • Pre competition fasting and spitting as needed	• Low fiber (<10g/day) diet for 3−4 days pre competition (ensure habitual fiber intake > 30g/day) • Low carbohydrate (<50g/day) diet for 4−7 days pre competition (ensure adequate habitual carbohydrate intake) • 90−120 mL/kg body mass/day 76-24h pre weigh in, 24 h pre weigh in 5−10 mL/kg body mass/day, sodium restriction (<1000 mg/day) 24 h pre weigh in • If needed completely cease food and fluid intake 1-6h pre weigh in • If needed stimulate saliva flow via chewing gum or spice while spitting into a cup 30-60min pre weigh in or after first unsuccessful weigh in attempt
>5%	• None	• Change weight class • Continue habitual diet and fluid intake

In summary, SBM can facilitate competing in a lower weight class and improve relative performance. However, it must be individualized and well justified. A data-driven, conservative, and ethical approach is essential. Rehydration and refueling between weigh-in and competition are critical. SBM should be targeted and strategic, not routine. Ongoing athlete education, systematic monitoring, and incorporation of emerging research are vital for safe and effective SBM in powerlifting and related strength sports.

## Author contributions

None.
